# Metformin exacerbates diabetic amyotrophy via oxidative stress and gut microbiota alterations

**DOI:** 10.3389/fmicb.2026.1778515

**Published:** 2026-04-13

**Authors:** Hejie Wang, Zhigang Cao, Wafa Yousaf, Abdul Haseeb, Ziyang Wang, Jiangang Zheng

**Affiliations:** 1 School of Public Health, Changzhi Medical College, Changzhi, Shanxi, China; 2 Laboratory of Environmental Factors and Population Health, Shanxi Higher Education Institutions of Science and Technology Innovation Plan Platform, Changzhi, China; 3 Key Laboratory of Environmental Pathogenic Mechanisms and Prevention of Chronic Diseases, Changzhi Medical College, Changzhi, China; 4 College of Veterinary Medicine, Shanxi Agricultural University, Taigu, Shanxi, China; 5 Institute of TCM, Xinjiang Medical University, Urumqi, Xinjiang, China

**Keywords:** *Desulfovibrio*, diabetic amyotrophy, metformin, neuronal nitric oxide synthase, oxidative stress

## Abstract

**Objective:**

This study aims to explore the targets and mechanisms by which metformin (MET) exacerbates diabetic amyotrophy (DA) using a streptozotocin (STZ)-induced diabetic SD rat model.

**Method:**

The targets of MET were acquired from 10 databases, including *HIT* and *TCMSP*. The DA targets were obtained from the *GeneCards* database. The associated targets were imported into the Venn analysis platform to draw Venn diagrams, and their intersections were visualized. A target protein–protein interaction (PPI) network was constructed using the Search Tool for the Retrieval of Interacting Genes/Proteins (STRING) and Database for Annotation, Visualization, and Integrated Discovery (DAVID) databases and Cytoscape software, and the core targets were identified. GO enrichment and KEGG pathway analyses were performed on the targets, and the construction of the “MET exacerbated DA target-pathway” network was carried out. Molecular docking was performed using Schrödinger Maestro 13.5 software to determine the binding free energy and binding mode of MET with the target protein. The expression of NOS1 and 3-nitrotyrosine proteins in gastrocnemius muscle tissue was detected by WB. The expression of 3-nitrotyrosine protein in gastrocnemius muscle tissue was detected using an enzyme-linked immunosorbent assay (ELISA). The localization of NOS1 in the gastrocnemius muscle tissue was detected using immunohistochemistry (IHC). 16S rDNA gene sequencing was performed to detect structural changes in the fecal microbiota.

**Results:**

Network toxicology analysis revealed 19 intersecting proteins. GO functional enrichment and KEGG pathway analyses demonstrated that the obtained targets were involved in molecular functions, biological processes, and cellular components and were involved in pathways such as drug metabolism—other enzymes, peroxisomes, and nucleotide metabolism. Based on the crystal structures of potential target proteins, complex structural models of target–MET were constructed through molecular docking (extra precision [XP] mode of flexible docking), followed by MM-GBSA analysis. The IHC results indicated that MET significantly promoted the redistribution of NOS1 from the membrane region to the cytoplasm. WB results showed that MET significantly (*p* < 0.001) promoted the expression of 3-nitrotyrosine in the diabetic SD rat model but had no significant effect on the expression of NOS1. ELISA results demonstrated that MET significantly (*p* < 0.001) promoted the expression of 3-nitrotyrosine in the diabetic SD rat model. The 16S rDNA gene sequencing results showed that MET led to a significant (*p* < 0.05) increase in the abundance of *Desulfovibrio* in the intestinal tract of rats.

**Conclusion:**

MET exacerbates diabetic amyotrophy by promoting the increased relative abundance of *Desulfovibrio*, inducing muscle oxidative stress, and causing NOS1 dysregulation.

**Figure:**
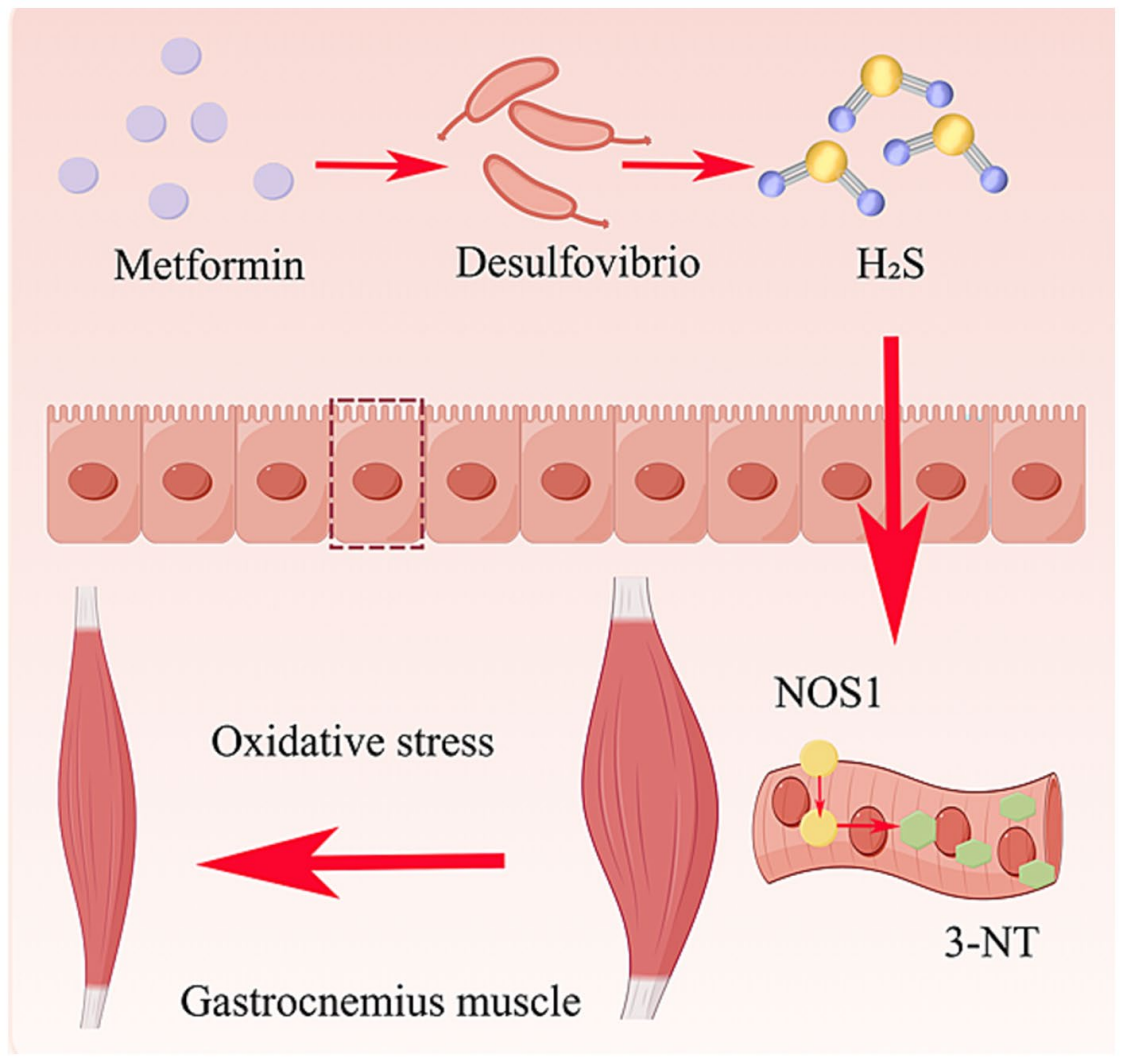

## 1 Introduction

Diabetic amyotrophy, a complication of diabetes, has been investigated for over a hundred years to explore its clinical characteristics and pathogenesis. In 1890, German neuropathologist Bruns first systematically described this syndrome. In the 1950s, Garland officially proposed the term “diabetic muscular atrophy” (Garland, 1955). This condition is typically characterized by unilateral pain of the thigh or buttocks at the onset. As the illness progresses, due to the multifocal involvement of the lumbosacral root, nerve plexus, and peripheral nerves, pain gradually disseminates to the contralateral leg, eventually leading to muscle atrophy and reduction in the patient’s quality of life (Garland, 1955). The pathogenesis of this disease may be closely related to the persistent hyperglycemic state caused by glucose metabolism disorders (Bowerman et al., 2012; Stapleton et al., 2014).

Metformin (MET), a fundamental drug used worldwide for the treatment of type 2 diabetes mellitus (T_2_DM), is clinically used for the management of T_2_DM, and its core pharmacological effects are centered on the regulation of cellular energy metabolism (Wise, 2016). Metformin weakens the glycemic effect of glucagon by inhibiting mitochondrial complex I and the glucagon-mediated cyclic adenosine monophosphate (cAMP)-protein kinase A signaling pathway and can also inhibit liver glucose production to exert a hypoglycemic effect (Farahani et al., 2025). For instance, the concentration of MET can clinically inhibit hepatic gluconeogenesis both *in vitro* and *in vivo* (LaMoia and Shulman, 2021). It can enhance the secretion of glucagon-like peptides through the synergistic effect of the insulin and Wnt signaling pathways, thereby achieving an antagonistic effect against glucagon (Kim et al., 2014). However, the risk of lactic acidosis in MET users is higher than that in other hypoglycemic drug users, indicating that MET has an associated usage risk (Li et al., 2017).

The pharmacological effects of MET are complex, and its biological effects are multifaceted. MET can indirectly promote tumorigenesis by reducing insulin levels and can directly act on tumor cell instability by inducing energy stress (El-Mir et al., 2008). In the field of neuroprotection, when MET is used in combination with cyclosporine A (CsA), a classic inhibitor of protein tyrosine phosphatase (PTP), this combination can strongly alleviate neuronal apoptosis, demonstrating its potential as a treatment for diabetes-related neurodegenerative diseases (El-Mir et al., 2008; Ullah et al., 2012). MET alleviates muscle atrophy induced by injury and obesity (Hasan et al., 2019; Yoon et al., 2025). However, MET can also regulate myostatin in skeletal muscle cells through the AMPK-FoxO3a-HDAC6 axis, thereby impairing muscle function (Kang et al., 2022). This indicates that MET has adverse effects on muscle health, which may depend on the dosage and treatment duration.

The gut microbiota participates in the development of T_2_DM and prediabetes through metabolites; approximately 70% of cases are related to poor diet, disrupting the microbiota (Wu H. et al., 2025). MET can alter the composition of the gut microbiota by increasing *Akkermansia muciniphila* (mucoprotein degradation) and various short-chain fatty acid (SCFA)-producing microbiota, and an increase in SCFA-producing bacteria (butyric acid and propionic acid) can improve blood glucose levels (Forslund et al., 2015). This indicates that the regulation of intestinal flora by MET appears to be beneficial to the human body; however, there are relatively few studies on the negative regulation of intestinal flora by MET.

Although the effectiveness of MET in T_2_DM has been confirmed in research and clinical practice, the results of our study indicate that high-dose MET can promote diabetic muscular atrophy (Wang et al., 2025). Consequently, our study used network toxicology, computational biology, and STZ-induced diabetes models in SD rats to explore the targets and mechanisms by which MET promotes diabetic amyotrophy, thereby providing a theoretical foundation for the safe clinical use of MET.

We therefore hypothesize that metformin exacerbates diabetic amyotrophy by reshaping gut microbiota (especially by increasing the abundance of *Desulfovibrio*) to trigger systemic oxidative stress, which further causes subcellular mislocalization and functional dysregulation of NOS1 in the gastrocnemius muscle, ultimately promoting muscle damage.

## 2 Materials and methods

### 2.1 Compounds and antibodies

MET (Cat: HY-B0627), Streptozotocin (STZ, Cat: HY-13753), and Dapagliflozin (Cat: HY-10450) were purchased from MCE (USA) with purities of 99.79, 99.98, and 99.97%, respectively. An ELISA kit was purchased from Jiancheng (China, Cat: H394-1-1).

Anti-NOS1 (Cat: GB15145) rabbit polyclonal antibody (pAb), anti-*β*-actin (Cat: GB15003) rabbit mAb, and goat anti-rabbit secondary antibody (Cat: GB23303) were purchased from Servicebio (Wuhan Servicebio Technology Co., Ltd., Wuhan, China). Anti-3-nitrotyrosine (Cat: bs-8551R) rabbit pAb was purchased from Bioss (Beijing, China).

### 2.2 Animal modeling and treatment

Four-week-old male SD rats (weight: 100 g) were procured from SPF Biotechnology Co., Ltd. (Beijing, China) and were equally distributed among the groups. The animals were maintained in a controlled environment with a 12-h light/dark cycle and free access to food and water. Animal experiments were approved by the Ethics Committee of Changzhi Medical College (DW2024140, July 22, 2024) and conducted under its guidance and regulations. After a one-week acclimatization period, the rats were randomly allocated into four groups (n = 8 per group): (1) Control group (Con); (2) Diabetic model group (DM): Rats were fed a high-sugar and high-fat diet (containing 67% maintenance diet, 10% lard, 20% sucrose, 2.5% cholesterol, and 0.5% sodium cholate) over 4 weeks, subsequently fasted for 12 h, and then administered a single intraperitoneal injection of STZ at 45 mg/kg (Wang et al., 2025); (3) Metformin group (MET): After 2 weeks of STZ injection, 500 mg/kg MET was administered by gavage for 4 weeks. (4) Dapagliflozin group (DAPA): After 2 weeks of STZ injection, 1 mg/kg dapagliflozin was administered by gavage for 4 weeks. STZ (9 mg/mL) was dissolved in 0.9% saline solution. A volume of 0.5% carboxymethyl cellulose (Solarbio, China, Cat: C8620) was used to dissolve the doses of MET (100 mg/mL) and DAPA (0.25 mg/mL). The control and model groups were administered an intraperitoneal injection and oral gavage of 0.9% saline and 0.5% carboxymethyl cellulose, equal to the treatment and positive control groups. The rats were euthanized with CO_2_, and the gastrocnemius muscle tissues were dissected, fixed, and frozen. The parameters of CO_2_ euthanasia were as follows: the chamber volume was 50 × 37 × 28 cm, the flow rate was 30% of the chamber volume/min, the CO_2_ concentration was gradually increased to 90% of the chamber volume, and the treatment time was 5 min (Wang et al., 2025).

### 2.3 Network toxicology

#### 2.3.1 Screening of MET and DA-related targets

“MET” and “Diabetic Amyotrophy” were used as search terms to collect relevant targets. First, the protein structure, compound CID, MF, and SMILES of MET were downloaded using the *PubChem* database.^1^ Through *HIT*,^2^ *TCMSP*,^3^ *BATMAN*,^4^ *ITCM*,^5^ *NAPSS*,^6^ *TCMSID*,^7^ HERB,^8^ SwissTargetPrediction,^9^ *CHEMB*L,^10^ *SymMap*,^11^ and *PharmMapper* database^12^ accessed the MET’s target collection. Finally, we logged the data into the *GeneCards* database^13^ to retrieve target data related to DA.

#### 2.3.2 MET promotes the visualization analysis of DA targets and the construction of PPI networks

Screening MET-related targets associated with DA targets was imported into the Venny platform,^14^ mapped Wayne, and visualized as an intersection to obtain MET-exacerbated DA targets. Then, the target was imported into the *STRING*^15^ database, and the species “*Homo sapiens*” was selected to obtain the interaction relationship of the target protein, and the PPI interaction graph and TSV files were exported. The TSV file was imported into Cytoscape 3.9.1 software to draw the Hub Network. The Network Analyzer tool was used to conduct a topological analysis of the network. Node size and color depth were used to reflect the degree of size, and edge thickness was used to reflect the size of the combined score. Simultaneously, core targets were screened based on the values of node connection (degree), node betweenness, and node closeness.

#### 2.3.3 GO enrichment and KEGG pathway analysis

We logged in to the DAVID database,^16^ imported the potential targets for MET exacerbated-DA, and selected the species “*homo sapiens*.” The selected identifier was set, and the types of official gene symbols and genes were listed. GO functional and KEGG pathway enrichment analyses of the potential targets of MET-exacerbated DA were performed, and the results were exported as a TXT file. The number of targets was arranged in descending order for GO (Biological Processes, Molecular Functions, and Cellular Components) and KEGG enrichment. Using the top 20 *p*-value targets, the data were imported into the microscopic letter online mapping platform,^17^ a map column type, and a bubble chart.

#### 2.3.4 Construction of toxic-target-pathway network

Cytoscape 3.9.1^18^ software was used to import the network file and type file of MET-core target-pathway, respectively; construct the “treatment-target-pathway” network diagram and improve it using layout and style tools.

### 2.4 Molecular docking

#### 2.4.1 PDB ID (protein data Bank ID)

NOS1: https://www.rcsb.org/structure/6CID (PDB ID: 6CID Chain A). NOS3: https://www.rcsb.org/structure/4D1P (PDB ID: 4D1P Chain A). NF-κB1: https://www.rcsb.org/structure/8TQD (PDB ID: 8TQD). PLAU: https://www.rcsb.org/structure/2O8T (PDB ID: 2O8T). PNP: https://www.rcsb.org/structure/4EAR (PDB ID: 4EAR Chain A). DPYD: Prediction of protein crystal structure using AlphaFold3. HEXA: https://www.rcsb.org/structure/2GJX (PDB ID: 2GJX Chain A). IDO1: https://www.rcsb.org/structure/8ABX (PDB ID: 8ABX). NOS2: https://www.rcsb.org/structure/3E7G (PDB ID: 3E7G Chain A). EGFR: https://www.rcsb.org/structure/8A27 (PDB ID: 8A27). HEXB: https://www.rcsb.org/structure/1NOW (PDB ID: 1NOW Chain A). XDH: https://www.rcsb.org/structure/2E1Q (PDB ID: 2E1Q Chain A). ACHE: https://www.rcsb.org/structure/4M0E (PDB ID: 4M0E Chain A).

#### 2.4.2 Protein preprocessing

The crystal structures of the 13 core proteins were obtained from the *RCSB PDB* database.^19^ The protein preparation wizard module in the Schrödinger software was used to process the obtained protein crystals (protein preprocessing, regeneration of states of native ligand, H-bond assignment optimization, protein energy minimization, and removal of water ions).

#### 2.4.3 Ligand preprocessing

The 2D sdf structure file of MET was processed using the LigPrep module in Schrödinger, and all its 3D chiral conformations were generated.

#### 2.4.4 Active site recognition

The SiteMap module in Schrödinger was used to predict the best binding site. Subsequently, the most appropriate enclosing box was set in the Receptor Grid Generation module of Schrödinger to perfectly wrap the predicted binding sites, thereby obtaining the active sites of the 13 proteins.

#### 2.4.5 Molecular docking

Schrödinger Maestro 13.5 (February 2023 version) was used to perform molecular docking of the treated MET with the active sites of 13 core proteins, respectively (XP docking with the highest precision). The lower the score, the lower the binding free energy of MET and proteins, and the higher the binding stability.

#### 2.4.6 Molecular mechanics generalized born surface area (MM-GBSA) analysis

According to the MM-GBSA analysis of MET and the active sites of 13 core proteins, MM-GBSA dG Bind can approximately represent the binding free energy between small molecules and proteins. The lower the binding free energy, the higher the binding stability of the ligand compound to the protein.

### 2.5 Western blot analysis (WB)

The different protein levels in the gastrocnemius tissue were detected by WB (Wang et al., 2025). The tissues were lysed with RIPA buffer (Beyotime, China) containing 1 mM protease inhibitor (Solarbio, China) and 1 mM phosphatase inhibitor (Solarbio, China), and the cells were collected using a cell scraper. Total tissue protein was extracted, and the protein concentration was determined using the BCA protein assay kit (Beyotime, China). An equal amount of cell lysate was separated on a 10% SDS-polyacrylamide gel and transferred to a polyvinylidene fluoride (PVDF) membrane according to the size of the gel. The membrane was blocked with Tris-buffered Tween 20 (TBST) with 5% non-fat dry milk (Merck, Germany) at 25 °C for 2 h. Subsequently, the membrane was incubated with the following primary antibodies overnight at 4 °C: *β*-Actin Mouse Monoclonal antibody (1:50000), NOS1, and 3-nitrotyrosine Rabbit Polyclonal antibody (1:1000). Then, the membrane was washed with TBST three times and incubated with goat anti-rabbit secondary antibodies (1:20000) at 25 °C for 2 h. Finally, the target protein was detected using an enhanced chemiluminescence system (Boster, China). Densitometric values of the protein bands were quantified using ImageJ software. Importantly, separate PVDF membranes were used for the detection of target proteins (NOS1 and 3-Nitrotyrosine) and the loading control (β-actin).

### 2.6 Enzyme-linked immunosorbent assay (ELISA)

Gastrocnemius muscle tissue was accurately weighed, and nine times the volume of homogenizing medium (0.9% physiological saline) was added at a ratio of weight (mg): volume (μL) = 1:9. The samples were mechanically homogenized in an ice-water bath to prepare a 10% homogenate solution. The mixture was centrifuged at 3000 rpm for 10 min, and the supernatant was collected for determination. A 100-μL aliquot of the appropriately diluted sample to be tested was added to the coated reaction wells. (At the same time, make blank wells and standard wells diluted in multiple ratios.) The plate was sealed with a sealing film and incubated at 37 °C for 1.5 h. The liquid was discarded, 300 μL of washing solution was added to each well, soaked for 2 min, patted dry on absorbent paper, and the same procedure was repeated five times. Next, 100 μL of diluted biotinylated antibody working solution was added to each well. The plate was sealed with a sealing film and incubated at 37 °C for 1 h. The liquid was discarded, and 300 μL of washing solution was added to each well, soaked for 2 min, patted dry on absorbent paper, and repeated five times. Next, 100 μL of the diluted enzyme conjugate working solution was added to each well. The plate was sealed with a sealing film and incubated at 37 °C in the dark for 30 min. The liquid was discarded, and 300 μL of washing solution was added to each well, soaked for 2 min, patted dry on absorbent paper, and repeated five times. TMB substrate solution (100 μL) was added to each well and incubated at 37 °C in the dark for 15 min until a distinct color gradient appeared in the wells containing the standard substance diluted in multiple proportions. Then, 100 μL of 2 M sulfuric acid was added to each reaction well, and the color changed from blue to yellow. Within 10 min, using the microplate reader Epoch (BioTek Instruments, Winooski, VT, USA) at 450 nm, the blank control wells were calibrated to zero, and the OD value of each well was measured.

### 2.7 Immunohistochemistry (IHC)

Tissue samples of the gastrocnemius muscle from SD rats were fixed with 4% paraformaldehyde solution (Beijing Solarbio Science & Technology Co., Ltd., Beijing, China). The fixed tissues were dehydrated using a fully automatic dehydrator (JT-12S, Wuhan Junjie Electronics Co., LTD., China), trimmed, embedded, and sectioned into paraffin sections. The expression of NOS1 in gastrocnemius muscle tissue was detected using immunohistochemistry (IHC). Paraffin sections were dewaxed in a water bath, followed by antigen retrieval, endogenous peroxidase blocking, and bovine serum blocking. The primary antibody of NOS1 (1:100) was applied, and the sections were incubated overnight in a 4 °C wet incubator, followed by the application of a secondary antibody (HRP-labeled goat against rabbit, 1:100). DAB drops were added to the tissue, and the color development time was controlled under a microscope. The positive slices turned brownish-yellow. The slices were rinsed with distilled water to block color development. They were re-stained with hematoxylin for 3 min and finally dehydrated and sealed (Wang et al., 2025). Images were acquired using a Pannoramic 250 digital section scanner (3DHISTECH, Hungary).

### 2.8 Sequencing analysis of 16S rDNA intestinal microbiota structure spectrum

Sequencing analysis of the structural spectrum of the 16S rDNA intestinal microbiota was performed using the Illumina MiSeq sequencing platform. Using the paired-end sequencing method, small fragment libraries were constructed for sequencing. The optimized sequence (tags) was obtained by filtering the original sequence and performing double-ended splicing. The optimized sequence was clustered and divided into OTUs, and their species classification was obtained based on the sequence composition of the OTUs. Based on the OTU analysis results, further *α*-diversity analysis, *β*-diversity analysis, LEfSe analysis of significantly different species, and KEGG function prediction analysis were performed. The primers used for amplification in this experiment were the 16S rDNA (V3 + V4) region primers of bacteria: 341F: 5’-CCTACGGGNGGCWGCAG-3′ and 805R: 5’-GACTACHVGGGTATCTAATCC-3′. DNA extraction, PCR amplification, and sequencing were performed in collaboration with the Servicebio® Biotechnology Company.

### 2.9 Statistical analysis

All data were presented as mean ± SEM and repeated at least three times. Data were analyzed using GraphPad Prism™ software (GraphPad Software, Inc., California, USA), version 5.0. One-way analysis of variance (ANOVA), followed by Dunnett’s post-test, was used to determine the difference between the groups. All groups were compared to the model group; *p < 0.05, **p < 0.01, and ***p < 0.001.

## 3 Results

### 3.1 Network toxicology screening for targets of MET exacerbating DA

**Figure 1:**
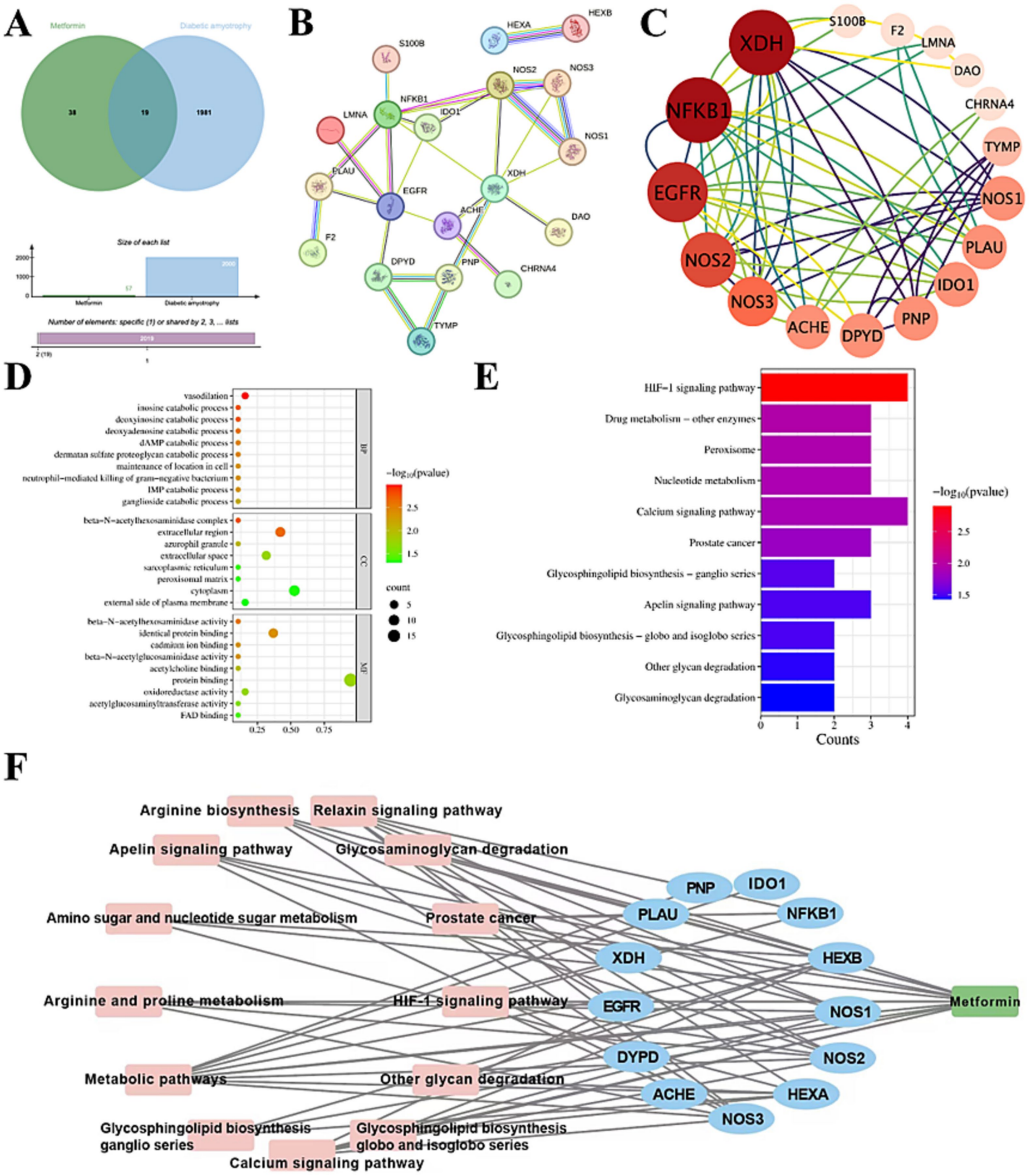
Network pharmacology predicts the targets of MET in exacerbating DA. **(A)** Venn diagram of potential targets of MET exacerbating DA. **(B,C)** MET exacerbating DA potential targets, PPI network, and hub network. **(D)** Targets that were enriched by the Gene Ontology of MET in exacerbating DA. **(E)** Targets were enriched by the KEGG pathway of MET in exacerbating DA. **(F)** MET-target-pathway network model.

A total of 57 MET targets and 2,000 DA-related targets were collected, with the species being human. The intersection of the collected targets was obtained, and the resulting proteins emerged as a potential target for MET to exacerbate DA. The targets of MET to exacerbate DA were visualized using a Venn diagram (Figure 1A). A total of 19 intersectinG proteins were identified, and the results are shown in Table 1.

**Table 1:** The target of metformin promotes DA.

Serial number	Gene	Uniprot ID	Name
1	DAO	P14920	D-amino-acid oxidase
2	NOS1	P29475	Nitric oxide synthase 1
3	NOS3	P29474	Nitric oxide synthase 3
4	NF-κB1	P19838	Nuclear factor NF-kappa-B1
5	TYMP	P19971	Thymidine phosphorylase
6	PLAU	P00749	Urokinase-type plasminogen activator
7	PNP	P00491	Purine nucleoside phosphorylase
8	DPYD	Q12882	Dihydropyrimidine dehydrogenase [NADP (+)]
9	HEXA	P06865	Beta-hexosaminidase subunit alpha
10	IDO1	P14902	Indoleamine 2,3-dioxygenase 1
11	NOS2	P35228	Nitric oxide synthase 2
12	S100B	P04271	Protein S100-B
13	EGFR	P00533	Epidermal growth factor receptor
14	HEXB	P07686	Beta-hexosaminidase subunit beta
15	F2	P00734	Prothrombin
16	XDH	P47989	Xanthine dehydrogenase
17	ACHE	P22303	Acetylcholinesterase
18	CHRNA4	P43681	Neuronal acetylcholine receptor subunit alpha-4
19	LMNA	P02545	Prelamin-A/C

The 19 intersection targets were imported into the STRING database to obtain a protein–protein interaction network diagram (Figure 1B). The TSV files of the intersection targets were imported into Cytoscape 3.9.1. The CytoNCA plugin was used to screen the core targets with average node connectivity (5.89), average node betweenness (0.02), and average node compactness (0.75), and the hub network was plotted (Figure 1C). As shown in the figure, the core targets of MET exacerbating DA are ACHE, EGFR, XDH, DPYD, PNP, NF-κB1, IDO1, PLAU, HEXA, HEXB, NOS2, NOS3, and NOS1.

The 19 selected intersection targets were input into the DAVID database for GO function and KEGG pathway enrichment analysis, and 50 GO and 11 KEGG enrichment results were obtained (Supplementary File 1). Among them, 33 biological processes were involved, which were mainly related to vasodilation, the catabolic process of inosine, and the catabolic process of deoxyinosine. Eight cellular components were mainly related to the *β*-N-acetylhexosaminase complex, extracellular regions, and Tianqing granule. There were nine molecular functions, mainly related to the activity of β-N-acetylhexosaminase, the activity of oxidoreductase, and the binding of flavin adenine dinucleotide. KEGG analysis predominantly highlighted the hypoxia-inducible factor 1 signaling pathway, drug metabolism-other enzymes, peroxisomes, etc. Enrichment analysis of GO functions and KEGG pathway *p*-values in the top 20 results was mapped using the Microscopic Letter online platform^20^ generating column types and bubble charts (Figures 1D,E).

The 13 core targets of MET exacerbating DA and the top 20 pathways enriched with *p*-values were integrated using Cytoscape 3.9.1 software to construct the MET-target-functional network (Figure 1F). The nodes in the network graph represent MET, target proteins, and related pathways, and the edges represent the interactions between MET and specific proteins and pathways. There were 44 pairs of protein-pathway relationships.

### 3.2 The molecular docking results of MET with potential targets

**Figure 2:**
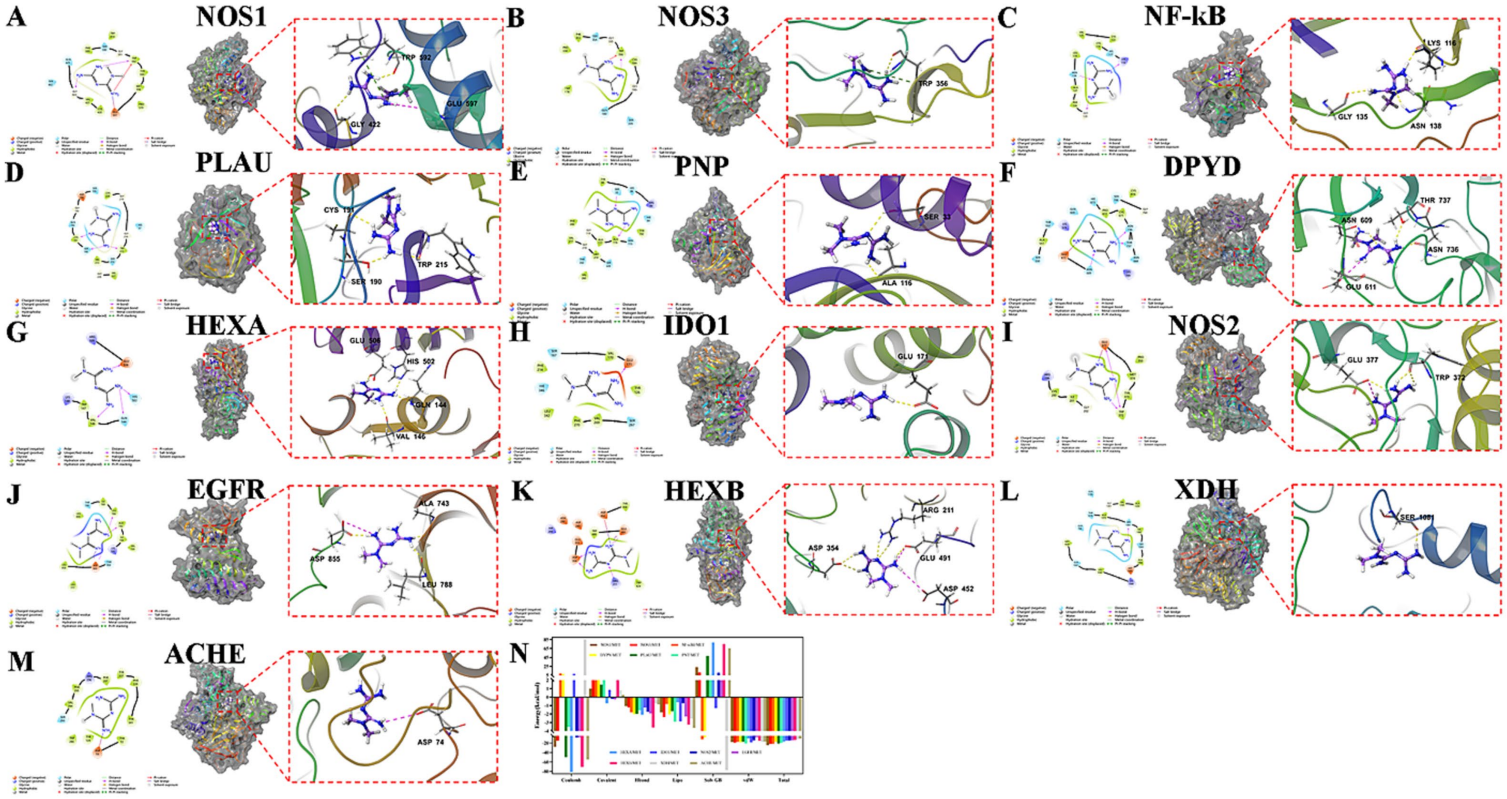
Molecular docking of complexes of MET with NOS1 **(A)**, NOS3 **(B)**, NF-κB **(C)**, PLAU **(D)**, PNP **(E)**, DPYD **(F)**, HEXA **(G)**, IDO1 **(H)**, NOS2 **(I)**, EGFR **(J)**, HEXB **(K)**, XDH **(L)**, and ACHE **(M)** (yellow indicates hydrogen bonds and green indicates *π*-cation bonds). **(N)** Statistical diagram of the MM/GBSA calculations for the complexes.

MET was attached to NOS1, NOS3, NF-κB1, PLAU, PNP, DPYD, HEXA, IDO1, NOS2, EGFR, HEXB, XDH, and ACHE surfaces. The NOS1 protein residues PHE589, TRP414, TRP592, TYR593, and MET594 exerted hydrophobic interactions on MET. This ligand formed a hydrogen bond with residue GLY422, a salt bridge with residue GLU597, and a hydrogen bond and *π*-cation bond with residue TRP592 (Figure 2A). The NOS3 protein residues PHE353, PRO334, TRP178, TRP356, and CYS184 exerted hydrophobic interactions on MET. This ligand formed a hydrogen bond and a π-cation bond with the residue TRP356 (Figure 2B). NF-κB1 protein residues, such as PHE136, ALA137, LEU139, VAL125, and CYS118, exerted hydrophobic interactions on MET. This ligand formed a hydrogen bond with residues LYS116, ASN138, and GLY135 (Figure 2C). The PLAU protein residues CYS220, CYS191, VAL213, VAL227, and TRP215 exerted hydrophobic interactions on MET. This ligand formed a hydrogen bond with residues CYS191, SER190, and TRP215 (Figure 2D). PNP protein residues, such as TYR88, PHE200, ALA117, ALA116, and TYR192, exerted hydrophobic interactions on MET. This ligand formed a hydrogen bond with residues SER33 and ALA116 (Figure 2E). The DPYD protein residues CYS816, CYS671, PRO672, and ALA551 exerted hydrophobic interactions on MET. This ligand formed a hydrogen bond with each of the residues ASN609, THR737, and ASN736 and a salt bridge with residue GLU611 (Figure 2F). The HEXA protein residues TRP147 and VAL146 exerted hydrophobic interactions on MET. This ligand formed a hydrogen bond with residues VAL146, GLN144, HIS502, and GLU506 (Figure 2G). Residues of the IDO1 protein, such as VAL170, TYR126, VAL269, PHE270, and LEU342, exerted hydrophobic interactions on MET. This ligand formed a hydrogen bond with the residue GLU171 (Figure 2H). The NOS2 protein residues PRO350, MET374, TYR373, TRP372, and ILE201 exerted hydrophobic interactions on MET. This ligand formed two hydrogen bonds with the residue TRP372 and one hydrogen bond and a salt bridge with the residue GLU377 (Figure 2I). EGFR protein residues, such as ILE789, LEU788, ALA743, ILE744, and VAL726, exerted hydrophobic interactions on MET. This ligand formed a hydrogen bond with residues LEU788 and ALA743, as well as a hydrogen bond and a salt bridge with residue ASP855 (Figure 2J). The HEXB protein residues TYR450, TRP489, and TRP424 exerted hydrophobic interactions on MET. The ligand formed interactions with ARG211 and ASP354. Each formed two hydrogen bonds: a salt bridge with residue ASP452 and a hydrogen bond and a salt bridge with residue GLU491 (Figure 2K). XDH protein residues, such as ALA1079, VAL1260, ALA1259, VAL1082, and ALA1084, exerted hydrophobic interactions on MET. This ligand formed a hydrogen bond with residue SER1081 (Figure 2L). The ACHE protein residues VAL294, PHE295, PHE297, TYR337, and PHE338 exerted hydrophobic interactions on MET, and the ligand formed a salt bridge with the residue ASP74 (Figure 2M).

Based on the comprehensive analysis of the XP docking (Table 2) and the MM-GBSA results (Figure 2N), the MET docking with NOS1 performed the best, with a docking score of −2.637, and the MM-GBSA result was −24.68 kcal/mol. The binding free energy was very low, and the docking score was relatively low, indicating that MET docking with NOS1 was the most stable compared to the other proteins.

**Table 2:** XP docking score of target with metformin.

Compound	Target	XP Gscore
Metformin	NOS1	−2.637
	NOS3	−2.478
	NF-κB1	−1.581
	PLAU	−2.764
	PNP	−3.152
	DPYD	−2.506
	HEXA	−2.776
	IDO1	−2.136
	NOS2	−3.033
	EGFR	−4.007
	HEXB	−3.215
	XDH	−2.4
	ACHE	−3.607

### 3.3 MET induces NOS1 dysregulation and promotes the expression of 3-nitrotyrosine

**Figure 3:**
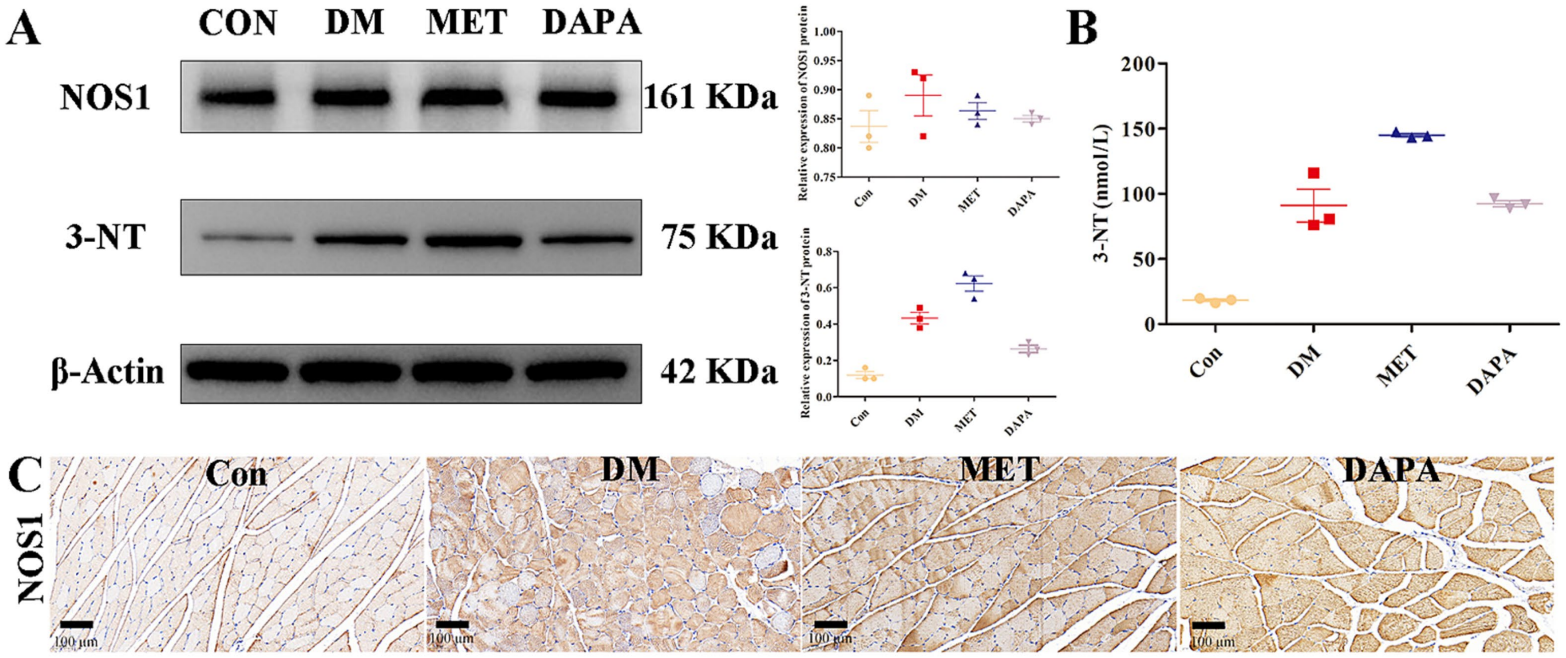
MET induces NOS1 dysregulation and promotes the expression of 3-nitrotyrosine. **(A)** The expression of NOS1 and 3-nitrotyrosine proteins was detected by western blot. The original blots are shown in Supplementary Figure 1. **(B)** The expression of 3-nitrotyrosine protein was detected by ELISA. **(C)** The expression of the NOS1 protein was detected by IHC after MET treatment.

The expression of NOS1 and 3-nitrotyrosine protein was detected by WB. Compared with the control group, the DM group significantly induced the production of 3-nitrotyrosine protein (*p* < 0.001) but did not affect the expression of NOS1 protein. Compared with the DM group, MET significantly induced the production of 3-nitrotyrosine protein (*p* < 0.001) but did not affect the expression of NOS1 protein (Figure 3A). The expression of 3-nitrotyrosine protein was detected by ELISA and was consistent with the WB results (Figure 3B). IHC results showed that MET promoted the transfer of NOS1 from the myometrium to the cytoplasm (Figure 3C).

### 3.4 Alpha diversity and ANOSIM analysis

**Figure 4:**
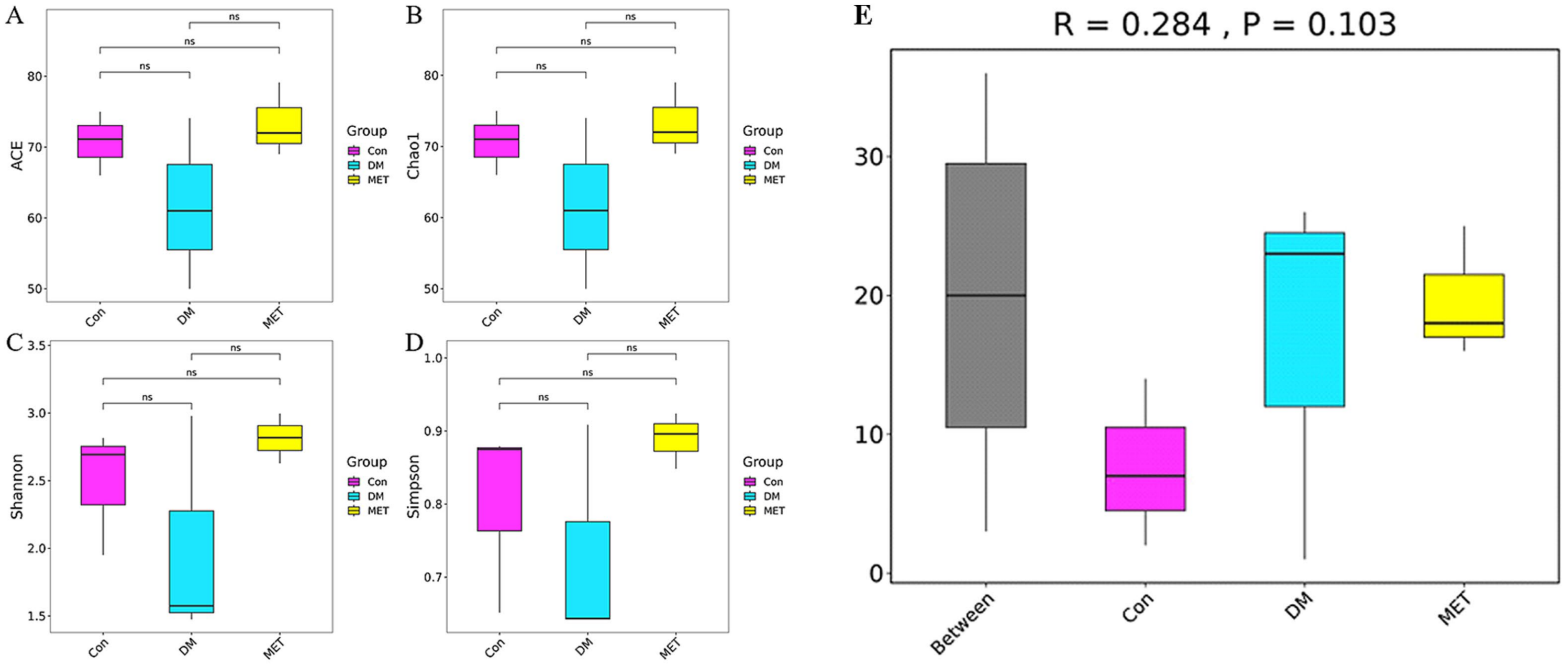
Results of *α*-diversity and ANOSIM analysis: **(A)** ACE index, **(B)** Chao1 index, **(C)** Shannon index, **(D)** Simpson index, **(E)** Results of the ANOSIM analysis.

The results of *α*-diversity analysis revealed that compared with the control group, there were no significant differences in abundance-based coverage estimator (ACE, Chao1, Shannon, and Simpson indices) in the DM group (*p* > 0.05). Compared with the DM group, there were no significant differences in ACE, Chao1, Shannon, and Simpson indices in the MET group (*p* > 0.05) (Figures 4A–D), indicating that neither DM nor MET treatment had a significant impact on the overall complexity and diversity structure of the intestinal microbiota in rats.

The ANOSIM analysis results showed that the R value of the difference between the groups was 0.284, and the *p* value was 0.103 (*p* > 0.05), indicating that there was no statistically significant difference in the intestinal flora structure among the groups. Specifically, compared to the control group, the microbiota structure of the DM group did not undergo significant changes. Compared to the DM group, there was no significant difference in the microbiota structure of the MET group (Figure 4E). This indicates that neither DM nor MET intervention had a significant impact on the overall structural composition of the intestinal microbiota in rats.

### 3.5 Lefse and KEGG analysis

**Figure 5:**
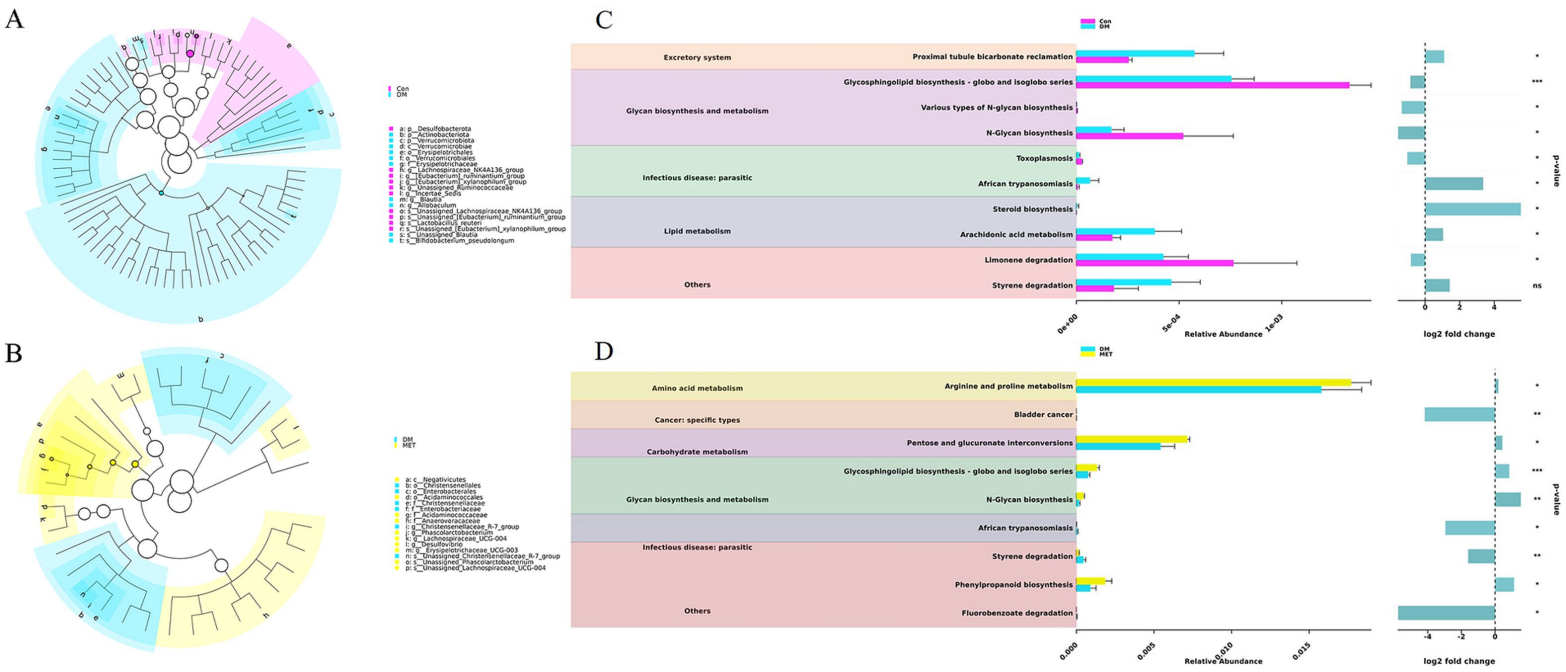
LEfSe and KEGG analyses. **(A)** Significantly different strains between the control and DM groups (*p* < 0.05, LDA > 2.0). **(B)** Significantly different strains between the DM and MET groups (*p* < 0.05, LDA > 2.0). **(C,D)** KEGG pathway analysis.

Although the α-diversity and ANOSIM analyses indicated that there were no significant differences in the overall microbial community structure, there were changes in specific taxa. The Lefse analysis results showed that compared with the control group, the abundance of bacteria such as *Desulfobacterota*, *Lachnospiraceae NK4A136*, *[Eubacterium] xylanophilum*, *Incertae Sedis*, and *Lactobacillus reuteri* in the DM group was significantly decreased (*p* < 0.05, LDA > 2.5). The abundance of bacteria such as *Actinobacteriota*, *Verrucomicrobiales*, *Erysipelotrichales*, *Blautia*, *Allobaculum*, and *Bifidobacterium pseudolongum* was significantly increased (*p* < 0.05, LDA > 2.5) (Figure 5A). Compared with the DM group, the abundance of bacteria, such as *Christensenellaceae*, *Enterobacteriaceae*, *Christensenellaceae R-7 group*, and *Unassigned Christensenellaceae R-7,* in the MET group was significantly decreased (*p* < 0.05, LDA > 2.5); the abundances of bacteria such as *Negativicutes*, *Acidaminococcales*, *Anaerovoracaceae*, *Phascolarctobacterium*, *Lachnospiraceae UCG-004*, *Desulfovibrio*, and *Erysipelotrichaceae UCG-003* were significantly increased (*p* < 0.05, LDA > 2.5) (Figure 5B). This indicates that diabetes can lead to a decrease in the abundance of intestinal probiotics, such as *Lactobacillus reuteri,* in rats. MET can exert therapeutic effects on diabetes through bacteria, such as *Lachnospiraceae UCG-004*, but it also leads to an increase in bacteria-related adverse effects associated with *Desulfovibrio*.

The KEGG analysis results demonstrated that proximal tubule bicarbonate reclamation, glycosphingolipid biosynthesis—globo and isoglobo series, African trypanosomiasis, arachidonic acid metabolism, limonene degradation, etc., were significantly (*p* < 0.05) enriched between the Con and DM groups (Figure 5C). Pathways such as arginine and proline metabolism, pentose and glucuronate interconversions, glycosphingolipid biosynthesis-globo and isoglobo series, African trypanosomiasis, and phenylpropanoid biosynthesis were all significantly (*p* < 0.05) enriched between the MET and DM groups (Figure 5D).

## 4 Discussion

Our study employed network toxicology, molecular docking, and molecular dynamics simulations to evaluate the role and potential mechanisms of MET in exacerbating DA. Initially, based on network toxicology analysis, NOS1 was identified as a key target for metformin (MET)-exacerbated diabetic amyotrophy (DA) in three core steps. First, 57 MET-related targets (from 10 databases, including HIT and TCMSP) and 2000 DA-associated targets (from GeneCards) were intersected via Venny, yielding 19 targets for MET exacerbating DA that were identified through network toxicology. Second, PPI network construction and topological analysis showed that NOS1 ranked among 13 hub targets due to its high connectivity and central regulatory role. Third, GO/KEGG enrichment confirmed that NOS1 was significantly involved in oxidative stress, nitric oxide biosynthesis, and skeletal muscle function pathways, which are closely aligned with the core mechanism of MET-exacerbated DA. Molecular docking is a widely used technology in drug development that is based on computer architecture. Among them, XP mode is a flexible docking (both the protein and the ligand are flexible); it is also a refined computational mode and can be used to perform higher-resolution molecular docking calculations for specific targets (Pillon et al., 2022). The XP docking results refer to the XP G-score. Generally, it is believed that if this value is < −6, it indicates that the ligand has a stable binding affinity with the protein. The analysis results of MM-GBSA are referred to as MM-GBSA dG_bind. When this value is lower than −30 kcal/mol, it indicates that the ligand is stably bound to the protein (Shi and Vanhoutte, 2017). The docking performance of MET with NOS1 was the best, with a docking score of −2.637 and an MM-GBSA result of −24.68 kcal/mol. The low binding free energy and docking score indicated that the binding of MET to NOS1 was stable. Therefore, in our study, NOS1 was identified as a potential targets of MET in exacerbating DA.

Oxidative stress, inflammation, mitochondrial dysfunction, reduced protein synthesis, and increased protein hydrolysis are key factors in muscle atrophy, particularly oxidative stress (Schiaffino et al., 2013; Yang et al., 2020; Zhang et al., 2023; Kanai et al., 2024). Oxidative stress is activated during the early stages of muscle atrophy. Hyperglycemia leads to excessive glucose metabolism, causing the mitochondrial respiratory chain to produce an excessive number of electron donors, resulting in the massive generation of reactive oxygen species (ROS), thereby causing muscle atrophy (Qiu et al., 2018). High levels of ROS oxidize and inhibit tyrosine phosphorylation of the insulin receptor substrate (IRS-1), hindering its downstream PI3K/Akt/mTOR pathway. The Akt/mTOR pathway is the most critical pathway for promoting muscle protein synthesis, and its inhibition directly leads to a reduction in muscle protein synthesis (Zhang et al., 2014; Li et al., 2025). Meanwhile, inhibited Akt cannot effectively phosphorylate and inhibit its downstream muscle atrophy-related gene, the FoxO transcription factor. The activated FoxO transcription factor upregulates two key muscle-specific E3 ubiquitin ligases, Atrogin-1/MAFbx and MuRF-1, leading to the degradation of myosin heavy chains and causing muscle atrophy. Oxidative stress can also activate autophagy, further accelerating muscle atrophy (Bentzinger et al., 2013).

NOS1 is a key enzyme that catalyzes the generation of nitric oxide from L-arginine and is located in the cell membrane of skeletal muscle cells (Umar and van der Laarse, 2010; Lechado et al., 2018). Under normal physiological conditions, low levels of nitric oxide (NO) produced by NOS1 are important signaling molecules that are crucial for maintaining muscle function and health (Baldelli et al., 2014). However, in an oxidative stress environment under pathological conditions, such as diabetes, the function of NOS1 becomes disordered and instead becomes an important factor that aggravates oxidative damage and promotes muscle atrophy. Oxidative stress consumes the essential cofactor NOS1—tetrahydrobiopterin (BH4). When BH4 is deficient, the molecular conformation of NOS1 changes, and it cannot normally catalyze L-arginine and oxygen to generate NO, which then falls away from the muscle membrane into the cytoplasm (Novoa et al., 2022). Superoxide anions produced by muscles react rapidly with residual NO to form more toxic peroxynitrite, further promoting diabetic amyotrophy. Among them, 3-nitrotyrosine is an important biomarker (Koppenol et al., 1992; Bandookwala and Sengupta, 2020). MET did not affect the total NOS1 protein level but induced a notable alteration in its subcellular localization, with a significant increase in cytoplasmic NOS1 and 3-NT levels. These qualitative IHC results imply that MET may trigger NOS1 dysregulation in terms of subcellular distribution rather than protein expression, which may further exacerbate muscle oxidative stress and contribute to diabetic amyotrophy.

Many studies show that MET can inhibit oxidative stress in skeletal muscles. MET can reduce oxidative stress in the body by lowering blood sugar levels. MET can significantly reduce oxidative stress levels in mouse skeletal muscles (Diniz Vilela et al., 2016). MET promotes the expression of NDUFA13 and mitochondrial biosynthesis through the AMPK signaling pathway, protecting cardiomyocytes cultured with high glucose from oxidative stress (Liu et al., 2020). Some studies have shown that MET can induce oxidative stress (Dixon et al., 2024). The results of our study are consistent with these findings, suggesting that MET regulates oxidative stress in a bidirectional manner, which may be closely related to its dose and duration of use. Our study further showed that high-dose MET treatment may cause abnormal localization of NOS1 by promoting oxidative stress, thus providing a new perspective for understanding the negative effects of MET in specific situations.

It is currently unclear how high-dose MET induces oxidative stress to exacerbate diabetic muscular atrophy. Studies have reported that intestinal metabolites can affect the hypoglycemic efficacy of MET, proving that the intestinal microbiota has a significant impact on the biological effects of MET (Koh et al., 2020). Given that the gut microbiota serves as a key hub for regulating the host metabolism and immune homeostasis, we further explored whether MET induces oxidative stress by altering the gut microbiota. The 16S rDNA sequencing results of the gut microbiota in our study indicated that MET treatment significantly increased the relative abundance of *Desulfovibrio*. *Desulfovibrio* is a sulfate-reducing bacterium and one of the symbiotic bacteria in the human gastrointestinal tract.

The terminal byproduct of its metabolic activity generates hydrogen sulfide (H_2_S) (Zhao et al., 2024). H₂S has a unique dual regulatory effect. At low concentrations, it can act as an effective antioxidant to reduce the damage caused by oxidative stress to cells and tissues. However, at high concentrations, H₂S may cause cytotoxicity and intensify oxidative stress responses (Cirino et al., 2023; Mallardi et al., 2025). *Desulfovibrio* can also induce oxidative stress in multiple organs (Wu R. et al., 2025). This finding contrasts with the beneficial effects of MET on the intestinal microbiota reported in the majority of studies, suggesting that MET may indirectly exacerbate oxidative stress through the negative regulatory pathway of enriching *Desulfovibrio*, thereby promoting abnormal NOS1 function and the progression of muscle atrophy.

This suggests that long-term or high-dose metformin treatment warrants closer monitoring for muscular complications, including muscle weakness, pain, and atrophy, in diabetic patients. Moreover, individual differences in gut microbiota composition may influence susceptibility to metformin-associated muscular adverse effects. Microbiota-targeted interventions or personalized dosage regimens may help mitigate metformin-induced muscle damage and improve the safety of metformin, particularly in diabetic patients. This study has several limitations. First, this study was conducted using an STZ-induced diabetic rat model, which may not fully recapitulate the complex pathophysiology of human type 2 diabetes and diabetic amyotrophy. Second, this study focused on the gastrocnemius muscle and fecal microbiota, and the findings may not represent all skeletal muscle groups or other tissue-specific effects. Finally, the causal relationship among metformin, *Desulfovibrio* overgrowth, oxidative stress, and NOS1 mislocalization has not been fully validated by intervention studies.

## 5 Conclusion

The disorder of NOS1 localization and accumulation of peroxynitrite were associated with MET treatment, which may be linked to the exacerbation of diabetic amyotrophy. Additionally, the increased abundance of *Desulfovibrio* may be associated with this phenomenon. However, it is important to note that the proposed mechanistic pathway connecting MET, gut microbiota changes, oxidative stress, and NOS1 dysregulation remains largely speculative. The current data primarily demonstrate correlations rather than direct causal relationships. Therefore, these findings should be interpreted with caution, and further studies are required to validate the underlying mechanisms. These findings offer a preliminary theoretical framework that warrants further investigation to guide the safe clinical application of MET.

## Data Availability

The datasets presented in this study can be found in online repositories. The names of the repository/repositories and accession number(s) can be found in the article/Supplementary material.

## Footnotes

1
https://pubchem.ncbi.nlm.nih.gov/

2
http://hit2.badd-cao.net/

3
https://old.tcmsp-e.com/

4
http://bionet.ncpsb.org.cn/batman-tcm/

5
http://itcm.biotcm.net

6
https://bidd.group/NPASS/

7
https://tcm.scbdd.com/home/index/

8
http://herb.ac.cn/

9
http://swisstargetprediction.ch

10
https://www.ebi.ac.uk/chembl/

11
http://www.symmap.org/

12
http://lilab-ecust.cn/pharmmapper/index.html

13
https://www.gene-cards.org

14
https://bioinfogp.cnb.csic.es/tools/Venny/index.HTML

15
https://string-db.org/

16
https://david.ncifcrf.gov/

17
http://www.bioinformatics.com.cn/

18
https://cyto-scape.org/

19
https://www.rcsb.org/

20
http://www.bioinformatics.com.cn/
