# Supplementary figures and images for: Metformin exacerbates diabetic amyotrophy via oxidative stress and gut microbiota alterations
Source: Front Microbiol. 2026 Apr 13;17:1778515. doi: 10.3389/fmicb.2026.1778515 (PMC13111326; doi:10.3389/fmicb.2026.1778515)

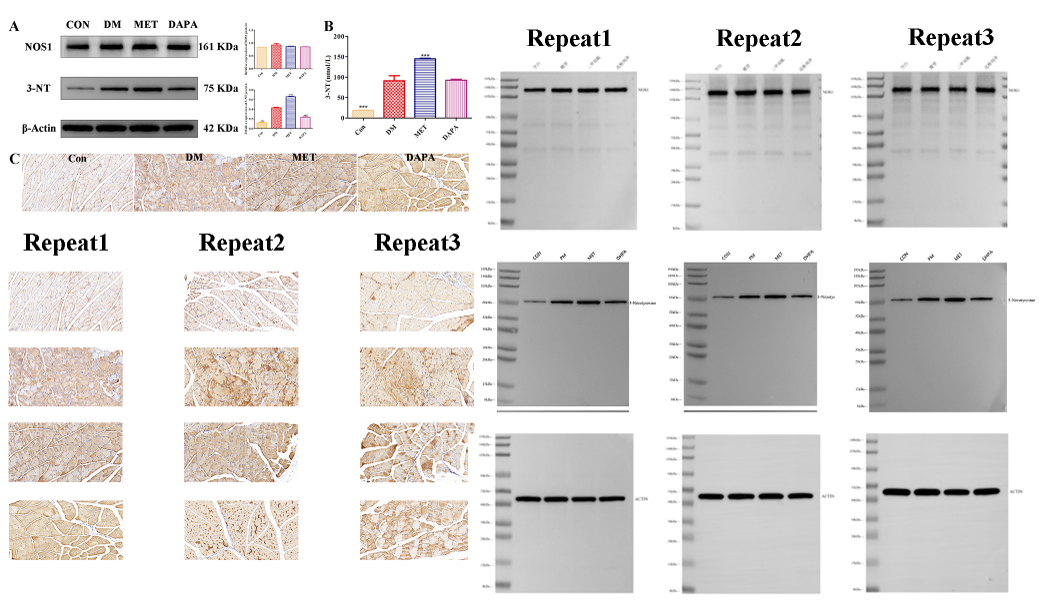

Supplement: Supplementary file 2 [file Image_1.tif]
